# The Effect of Advancing Age and Intraocular Pressure Injury on Retinal Ganglion Cell Function and Synaptic Connectivity

**DOI:** 10.1111/acel.70005

**Published:** 2025-02-27

**Authors:** Vicki Chrysostomou, Sevannah Ellis, Lewis E. Fry, Robert J. Hatch, Eamonn T. Fahy, Katharina C. Bell, Ian A. Trounce, Peter van Wijngaarden, Steve Petrou, Jonathan G. Crowston

**Affiliations:** ^1^ Centre for Vision Research Duke‐NUS Medical School Singapore Singapore; ^2^ Singapore Eye Research Institute Singapore Singapore; ^3^ The Florey Institute of Neuroscience and Mental Health The University of Melbourne Melbourne Victoria Australia; ^4^ Centre for Eye Research Australia, Royal Victorian Eye and Ear Hospital The University of Melbourne East Melbourne Victoria Australia; ^5^ NHMRC Clinical Trial Centre University of Sydney Camperdown New South Wales Australia; ^6^ Ophthalmology, Department of Surgery The University of Melbourne Melbourne Victoria Australia; ^7^ Save Sight Institute, Faculty of Medicine University of Sydney Camperdown New South Wales Australia

**Keywords:** aging, glaucoma, intraocular pressure, retinal ganglion cell, synapses

## Abstract

Age and elevated intraocular pressure (IOP) are the two major risk factors for developing glaucoma, a leading cause of blindness worldwide that is characterized by the loss of retinal ganglion cells (RGCs). Although vision loss is irreversible over the long term, accumulating evidence points to short‐term improvement of vision in glaucoma patients in response to certain interventions, suggesting that RGCs have the capacity to recover function. In the present study, we sought to investigate the mechanisms underlying loss and recovery of RGC function in response to aging and IOP injury, with a focus on synaptic connectivity. Using electroretinography, we found that advancing age was associated with a substantial reduction in function across all retinal layers in the absence of significant cell loss. A superimposed injury induced by IOP elevation led to the selective loss of RGC function in young and middle‐aged mice that was associated with a decrease in paired excitatory synapses. RGC functional recovery after injury was significantly delayed in middle‐aged mice and was mediated through different cellular mechanisms than in young mice. Whereas young mice regained excitatory synaptic inputs from bipolar cells, functional recovery in older mice was instead mediated through an increase in intrinsic RGC excitability, associated with modulation of the action potential threshold and axon initial segment length. Our findings provide new insights into the impact of advancing age on RGC resilience to IOP injury. Boosting the capacity for RGC recovery by reversing the effect of advancing age offers a new therapeutic approach for glaucoma management.

## Graphical abstract

The effect of advancing age and intraocular pressure injury on retinal ganglion cell function and synaptic connectivity. While young mice modulate their synaptic input to recover retinal ganglion cell function following injury, older mice compensate by altering their action potential threshold and axon initial segment (AIS) length to increase intrinsic excitability.

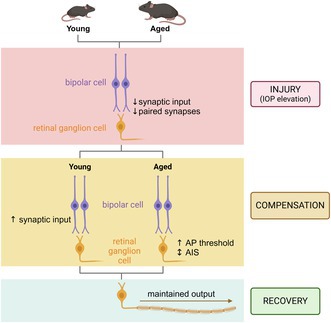

**Abbreviations**
AIS: axon initial segment
AP: action potential
eEPSC: evoked excitatory postsynaptic current
eIPSC: evoked inhibitory postsynaptic current
ERG: electroretinogram
IOP: intraocular pressure
pSTR: positive scotopic threshold response
RGC: retinal ganglion cell
VEP: visual evoked potential

## 1 Introduction

Vision loss from glaucoma results from the selective dysfunction and eventual death of retinal ganglion cells (RGCs) and constitutes a leading cause of global blindness. The main risk factors for developing glaucoma are advancing age and elevated intraocular pressure (IOP). Currently, lowering IOP remains the only treatment strategy for glaucoma. Although vision loss is irreversible over the longer term, there is accumulating evidence demonstrating short‐term visual recovery in response to the lowering of IOP in patients with glaucoma and in experimental animal models of elevated IOP (reviewed in Fry et al. 2018; Tang et al. 2020). Similar improvements in visual function have been demonstrated by the elevation of vitreous glucose (Casson et al. 2014) and metabolic supplements (De Moraes et al. 2022; Hui et al. 2020). Furthermore, inner retinal dysfunction has been shown to precede RGC loss and visual field defects by several years (Howell et al. 2007; Saleh, Nagaraju, and Porciatti 2007; Ventura et al. 2006). Early stages of RGC dysfunction may therefore provide a therapeutic window of opportunity where damage is reversible.

While the cellular mechanisms responsible for loss and recovery of RGC function in response to IOP elevation are not fully understood, synaptic and dendritic changes likely play a central role. A decrease in RGC dendritic arborisation and a loss of excitatory postsynapses is seen prior to significant cell death and optic nerve degeneration in mouse models of elevated IOP and in human glaucoma patients (Berry et al. 2015; Tribble et al. 2019; Williams et al. 2013). Recent data show that these changes can be reversed; insulin applied by topical instillation to the eye promoted RGC dendrite regeneration and restored excitatory synaptic inputs in a mouse model of chronic IOP elevation (El Hajji et al. 2024). Yet, several questions remain unanswered. Firstly, most experimental models have only investigated young animals so we do not know how advancing age, a major risk factor for glaucoma, alters RGC synaptic and dendritic responses to elevated IOP. Secondly, while previous studies have investigated changes in RGC synapse structure, the effects of IOP elevation on synapse function are still largely unknown. Finally, we do not know how synaptic and dendritic changes correlate with RGC output such as action potential firing or RGC‐derived signals of the full‐field electroretinogram, which is the most common means of assessing in vivo retinal function in the clinic and laboratory.

To address these knowledge gaps, we have used a well characterized mouse model of RGC injury following a time‐defined IOP challenge that induces loss and subsequent recovery of RGC function (Crowston et al. 2015). The rate of functional recovery in this model is highly dependent on mouse age. Furthermore, we have recently shown that older mice are unable to recover function after a repeat IOP challenge (Chrysostomou et al. 2024), suggesting that recovery mechanisms after a single injury are important in determining resilience to a second injury. In the current study, we first used full‐field electroretinography, visual evoked potentials and histology to characterize the effects of aging on ‐retinal function and cell survival up to 24‐months of age. We then used whole‐cell patch‐clamp electrophysiology to investigate changes in the functional excitatory synaptic connectivity and functional inhibitory synaptic connectivity of RGCs in response to a single IOP challenge; comparing these responses between young and middle‐aged mice. Finally, we used immunohistochemistry and high‐resolution microscopy to investigate changes in RGC synaptic and dendritic structure after an IOP challenge.

## 2 Results

### 2.1 Retinal Function Decreases With Advancing Age

**FIGURE 1:**
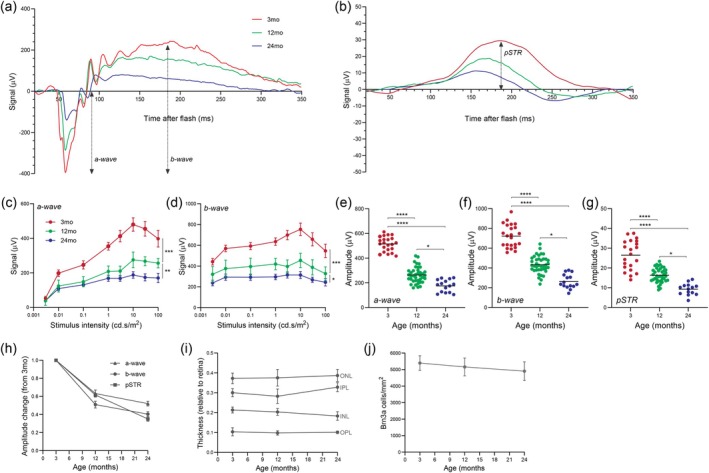
Decreased retinal function and stable cell numbers with advancing age in the C57BL/6J mouse. (a) Representative scotopic ERG responses demonstrating the a‐wave and b‐wave components in a mouse at 3‐, 12‐, and 24‐months of age. (b) Representative ERG responses demonstrating the pSTR waveform in a mouse at 3‐, 12‐, and 24‐months of age. (c, d) Luminance‐response functions for the a‐wave and b‐wave components of the scotopic ERG in 3‐, 12‐, and 24‐month‐old mice (*n* = 22 3‐month‐old mice; *n* = 40 12‐month‐old mice; *n* = 15 24‐month‐old mice). *p < 0.05, **p < 0.01, ***p < 0.001; two‐way ANOVA (stimulus intensity vs. age) with Tukey–Kramer post hoc test. (e) Maximum amplitudes of the a‐wave were elicited at 10 cd.s/m^2^, (f) b‐wave at 10 cd.s/m^2^ and (g) pSTR at 6.3e‐6 cd.s/m^2^. Solid black line represents the mean and each symbol represents a single animal (*n* = 22 3‐month‐old mice; *n* = 40 12‐month‐old mice; *n* = 15 24‐month‐old mice). *p < 0.05, ****p < 0.0001; one‐way ANOVA with Tukey–Kramer post hoc test. (h) Comparison of the rate of a‐wave, b‐wave and pSTR amplitude loss between 3 and 24 months of age. Amplitudes of each waveform are normalized to values from 3‐month‐old mice. (i) Thickness of cellular and plexiform layers measured on retinal cross‐sections taken from mice at 3‐, 12‐, and 24‐months of age. ONL: Outer nuclear layer, IPL: Inner plexiform layer, INL: Inner nuclear layer, OPL: Outer plexiform layer (*n* = 8 per age group). (j) RGC survival as quantified by the number of Brn3a‐labeled soma on retinal flatmounts (*n* = 9 3‐month‐old mice; *n* = 13 12‐month‐old mice; *n* = 4 24‐month‐old mice). Averaged data are presented as mean ± standard error of the mean.

We first assessed how retinal function changes with age in the C57BL/6J mouse by recording the full‐field flash electroretinogram (ERG) at 3‐, 12‐, and 24‐months of age under scotopic conditions. We did not observe any overt age‐related changes to corneal opacity or incidence of cataract in mice that may have hindered light transmission to the retina. ERG waveforms derived from photoreceptors (a‐wave), ON bipolar cells (b‐wave), and RGCs (pSTR) all showed a decrease in amplitude with advancing age (Figure 1a,b). Oscillatory potentials, which are present on the ascending limb of the b‐wave and thought to be generated by amacrine cells, also appeared to decrease in size in older mice although we did not quantify these changes. Amplitudes of the pSTR were measured in response to a single flash stimulus of 6.3e‐6 cd.s/m^2^, which coincides with the pSTR peak, while amplitudes of the a‐wave and b‐wave were measured in responses to a series of increasing stimulus intensities from 0.003 to 100 cd.s/m^2^ (Figure 1c,d). Analysis of the stimulus response curves showed a significant reduction in both a‐wave and b‐wave amplitudes between 3‐ and 12‐months of age (*p* < 0.001) and between 12‐ and 24‐months of age (*p* < 0.05). Interestingly, the characteristic decrease in a‐wave and b‐wave amplitudes that is seen after saturation in the stimulus response curves appeared to be blunted in 24‐month‐old mice. Figure 1e–g shows the maximum elicited amplitudes for each waveform at a given stimulus, plotted from individual mice. For all three waveforms, there was a significant difference in amplitudes between each age group studied (*p* < 0.05). Using these maximum values of amplitude, data were normalized to values obtained at 3‐months of age and the amplitude loss over time was plotted (Figure 1h). This analysis shows that there is greater loss of amplitude between 3‐ and 12‐months of age than between 12‐ and 24‐months of age for all three waveforms studied. Furthermore, Figure 1h shows that the reduction in b‐wave amplitude between 3‐ and 12‐months of age was significantly greater (*p* < 0.05) than the reduction in a‐wave amplitude (a‐wave 5.45% ± 2.6% vs. b‐wave 4.09% ± 2.7%), suggesting that loss of bipolar cell function was not purely consequent to a decrease in upstream signaling from photoreceptors. Between 12‐months and 24‐months of age, the degree of pSTR amplitude loss from RGCs (3.60% ± 0.83%) exceeded the equivalent loss of a‐wave signals from photoreceptors (1.47% ± 0.9%) and b‐wave signals from bipolar cells (1.75% ± 0.9%). This suggests that changes to the pSTR up to 12‐months of age could be purely consequent to a‐wave loss but beyond 12‐months could be RGC‐specific.

### 2.2 No Significant Loss of Retinal Neurons With Advancing Age

To accompany functional data, we assessed gross retinal structure and cell number in response to normal aging. Hoechst‐labeled retinal cross‐sections of eyes from 3‐, 12‐, and 24‐month‐old mice were used to quantify the thickness of the nuclear and synaptic layers. There was no significant change (*p* > 0.05) in the thickness of the outer nuclear layer, inner nuclear layer, outer plexiform layer or inner plexiform layer across age (Figure 1i). To assess RGC number, we immunolabeled flatmounted retinas with Brn3a, a marker capable of detecting significant decreases of RGC number with age and in response to IOP elevation (Meng et al. 2024). There was no significant change (*p* > 0.05) in the density of Brn3‐positive cells from 3‐ to 24‐months of age (Figure 1j). Collectively, these data suggest no substantial loss of retinal neurons with advancing age in the C57BL/6J mouse.

As advancing age and IOP elevation are known to be the two major risk factors for RGC loss in glaucoma, we next investigated the impact of age on functional recovery after an IOP challenge. In particular, we were interested to determine the electrophysiological characteristics of RGC function loss and recovery and whether this was different among young and older mice.

### 2.3 Recovery of Inner Retinal Function Following Intraocular Pressure Injury Is Delayed With Age

**FIGURE 2:**
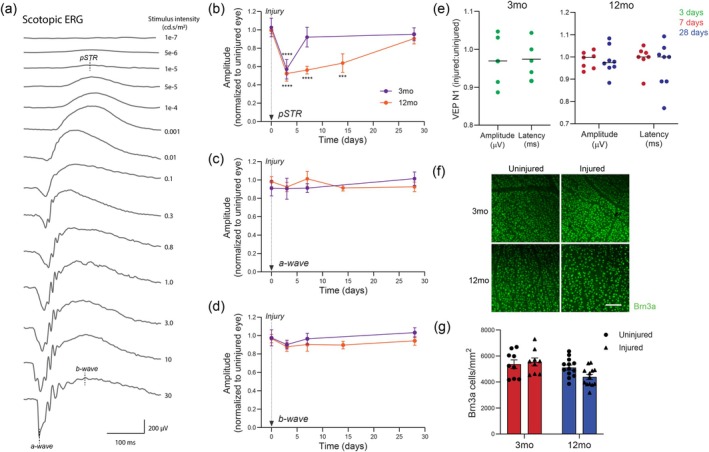
Aging slows retinal ganglion cell (RGC) functional recovery after intraocular pressure (IOP) injury. (a) Representative intensity series of ERG waveforms from a 3‐month‐old uninjured control eye, demonstrating the a‐wave (photoreceptor‐derived), b‐wave (bipolar cell‐derived) and pSTR (RGC‐derived) components. (b–d) ERG responses were recorded serially in 3‐ and 12‐month‐old mice at various intervals up to 28 days after IOP injury. Amplitudes in IOP‐elevated eyes were normalized to values from uninjured contralateral eyes. (*n* = 9 3‐month‐old mice; *n* = 13 12‐month‐old mice). ***p < 0.001, ****p < 0.0001 compared to day 0; one‐way ANOVA with Dunnett's *post hoc* test. (e) Flash visual evoked potentials (VEPs) were recorded to assess in vivo conduction of action potentials along the optic nerve. Values from IOP‐injured eyes were normalized to uninjured contralateral eyes. (*n* = 5 3‐month old mice; *n* = 8 12‐month‐old mice). (f) Representative micrographs showing immunohistochemical labelling of Brn3a‐positive cells on retinal flatmounts taken from eyes exposed to IOP elevation versus contralateral control eyes in 3‐ and 12‐month‐old mice. Scale bar: 50 μm. (g) RGC survival in response to IOP elevation was assessed by quantifying the number of Brn3a‐immunoreactive nuclei (*n* = 9, 3‐month‐old mice; *n* = 13, 12‐month‐old mice). Averaged data are presented as mean ± standard deviation.

We have previously established a model of acute IOP elevation that causes selective and reversible RGC dysfunction and allows investigation of RGC functional recovery following injury (Crowston et al. 2015). To more accurately determine age‐dependent differences in RGC recovery rates, here we analyzed pan‐retinal function in mice serially at 3, 7, 14, and 28 days post‐injury, which is a more comprehensive timeline than previously reported. Bilateral ERG responses to a series of stimulus intensities were recorded in each mouse (Figure 2a), and amplitudes from IOP‐elevated eyes were normalized to values from uninjured contralateral eyes. Confirming previous reports, IOP elevation caused a loss of RGC function manifest by a reduction in the pSTR component of the full‐field flash ERG (Figure 2b). In both 3‐and 12‐month‐old mice, IOP elevation induced a similar 50% reduction (*p* < 0.001) in pSTR amplitudes compared to baseline values at 3‐days post injury. The speed of functional recovery after IOP elevation, however, was highly dependent on mouse age. Younger 3‐month‐old mice recovered their pSTR amplitude to baseline levels by 7‐days and maintained this up to 28‐days post injury. RGC recovery in 12‐month‐old mice took significantly longer; pSTR amplitudes were persistently decreased at 7‐days (*p* < 0.0001) and 14‐days (*p* < 0.001) following IOP elevation and did not recover to baseline levels until 28‐days. Photoreceptor function, as measured by the relative a‐wave amplitude was unchanged following injury in 3‐ and 12‐month‐old mice (Figure 2c). Equally, bipolar cell function, as measured by b‐wave amplitude, was unchanged (Figure 2d). These findings support previous literature and the specificity of functional loss to the inner retina in this injury model.

We also assessed downstream RGC axonal function by recording flash visual evoked potentials (VEPs), which measure cortical activity in response to visual stimuli and provide a measure of optic nerve integrity (Ridder and Nusinowitz 2006). In both 3‐month‐old and 12‐month‐old mice, amplitude and latency of the N1 component of the VEP were unchanged following IOP elevation relative to uninjured eyes (Figure 2e). These results suggest that there was no major changes in axon conduction of action potentials and synaptic transmission after injury.

To estimate RGC death in response to IOP elevation we quantified the density of Brn3a immunoreactive nuclei on flatmounted retinas (Figure 2f). In 3‐month‐old mice, we did not detect any significant loss (*p* > 0.05) of Brn3‐positive cells at 7‐days post IOP elevation, a time when young mice had fully recovered pSTR amplitudes (Figure 2g). In 12‐month‐old mice, we assessed RGC loss at 28‐days post IOP elevation, which corresponds to full pSTR recovery in these older mice. Although there was a trend toward slightly lower density of Brn3a‐positive cells in injured eyes compared to fellow control eyes at 28‐days, this was not significant (14.14% decrease, *p* > 0.05; Figure 2g). We next went on to investigate whether there were any underlying differences in the mechanism of RGC recovery between the two ages of mice.

### 2.4 Young Mice Modulate Their Excitatory Input to Recover Retinal Ganglion Cell Function Following Intraocular Pressure Injury

**FIGURE 3:**
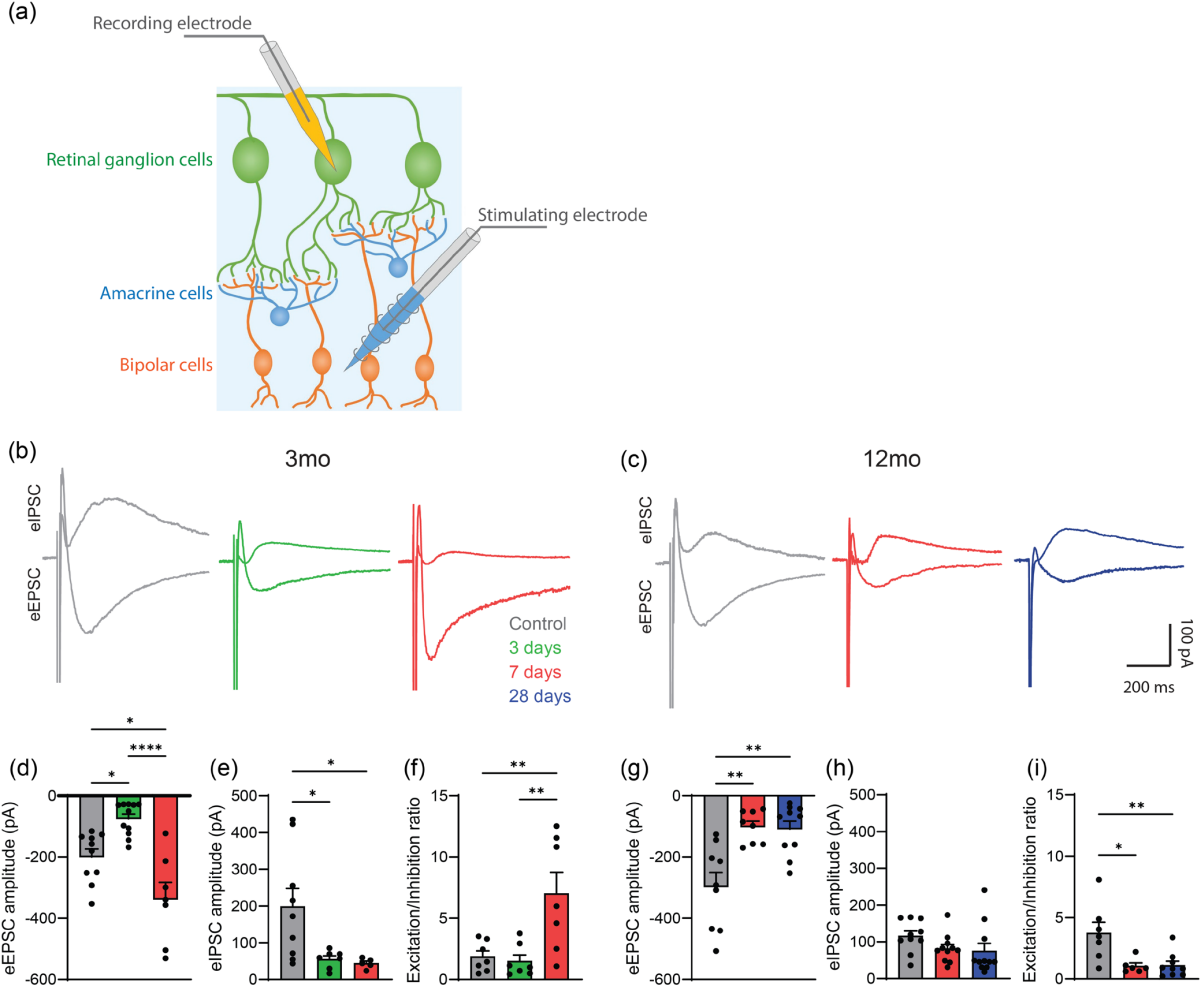
Young mice modulate their synaptic input to recover retinal ganglion cell (RGC) function following intraocular pressure (IOP) injury. (a) Schematic of whole‐cell patch‐clamp electrophysiology protocol. A dual electrode setup was used to stimulate local field potentials in the bipolar cell layer and record evoked excitatory (eEPSC) and inhibitory (eIPSC) postsynaptic currents from individual RGCs. (b, c) Example traces for amplitude of eEPSC and eIPSC recordings from RGCs in 3‐ and 12‐month‐old mice. In 3‐month‐old mice, traces were recorded from uninjured controls (gray) and at 3‐days (green) and 7‐days (red) after IOP elevation. In 12‐month‐old mice, traces were recorded from uninjured controls (gray), and at 7‐days (red) and 28‐days (blue) after IOP elevation. (d) Quantification of eEPSC amplitude and (e) and eIPSC amplitude in 3‐month‐old mice (*n* > 10 cells, 4 mice). (f) Excitation/inhibition ratio of eEPSC and eIPSCs from same cell in 3‐month‐old mice (*n* > 5 cells, 4 mice). (g) Quantification of eEPSC amplitude and (h) eIPSC amplitude in 12‐month‐old mice (*n* > 7 cells, 4 mice). (i) Excitation/inhibition ratio of eEPSC and eIPSCs from same cell in 12‐month‐old mice (*n* > 8 cells, 4 mice). *p < 0.05, **p < 0.01, ****p < 0.0001; one‐way ANOVA with Tukey–Kramer post hoc test. Averaged data are presented as mean ± standard error of the mean.

To investigate changes in the synaptic connectivity of RGCs, we used whole‐cell patch‐clamp electrophysiology in retinal flatmounts that were freshly isolated from mice at different intervals following IOP injury. We used a dual electrode setup where we stimulated local field potentials in the bipolar cell layer and recorded evoked excitatory (eEPSC) and inhibitory postsynaptic currents (eIPSC) from individual RGCs (Figure 3a–c). In 3‐month‐old mice, excitatory synaptic input in the form of eEPSC amplitude was significantly decreased by 62% (*p* < 0.05) at 3‐days post injury and not only recovered 7‐days post injury but increased significantly by 42% (*p* < 0.01) compared to uninjured controls (Figure 3d). Inhibitory synaptic input, as measured by eIPSC amplitude, was also significantly decreased by 69% (*p* < 0.05) at 3‐days post injury in 3‐month‐old mice and remained reduced at 7‐days post injury compared to uninjured controls (Figure 3e). This resulted in a significant increase (*p* < 0.01) in the excitation/inhibition ratio of RGCs 7‐days post injury compared to uninjured controls (Figure 3f). These data indicate that while IOP elevation induces a loss of evoked excitatory and inhibitory synaptic input, young mice appear to increase their excitation/ inhibition ratio, which may permit them to recover RGC function.

In 12‐month‐old mice, eEPSC amplitude was significantly decreased by 58% (*p* < 0.01) at 7‐days post injury, which is similar to the reduction seen in younger mice (Figure 3g). However, in contrast to the younger mice, eEPSC amplitude did not recover at 28‐days post injury in line with recovery of the ERG pSTR signal, suggesting an alternative mechanism. Inhibitory synaptic input as measured by eIPSC amplitude did not change from control values at either 7‐ or 28‐days post injury (Figure 3h), although we noted that eIPSC amplitude was already substantially reduced in 12‐month old controls compared to 3‐month old controls. Unlike in younger mice, 12‐month‐old mice had a significantly lower excitation/inhibition ratio at 7‐days (*p* < 0.05) and 28‐days (*p* < 0.01) post injury (Figure 3i). Together, these data indicate that 12‐month‐old mice do not recover RGC function by regaining excitatory inputs.

### 2.5 Older Mice Compensate Following Intraocular Pressure Injury by Increasing Intrinsic Retinal Ganglion Cell Excitability

**FIGURE 4:**
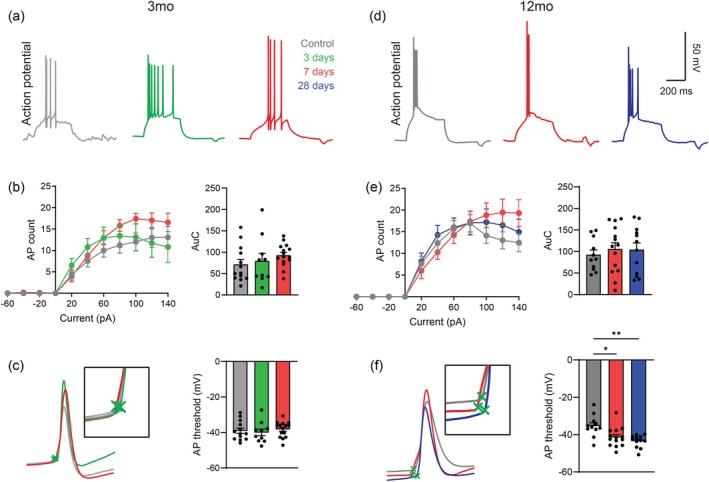
Older mice compensate following intraocular pressure (IOP) injury by increasing their intrinsic retinal ganglion cell (RGC) excitability. (a) Representative traces of action potential (AP) firing from RGCs in a 3‐month‐old mouse following injection of a rheobase current. Traces were recorded from uninjured controls (gray) and at 3‐days (green) and 7‐days (red) after IOP elevation. (b) AP input–output relationships and quantification of area under the curve (AuC) following injury in 3‐month‐old mice (*n* > 9 cells, 3 mice). (c) Representative traces of the first AP fired by RGCs at rheobase in a control 3‐month‐old mouse and following IOP elevation. Insert shows a close‐up of APs, where the green cross indicates the position of the AP threshold (*n* > 10 cells, 3 mice). (d) Representative traces of AP firing from RGCs in a 12‐month‐old mouse following injection of a rheobase current. Traces were recorded from uninjured controls (gray), and at 7‐days (red) and 28‐days (blue) after IOP elevation. (e) AP input–output relationships and quantification of area under the curve (AuC) following injury in 12‐month‐old mice (*n* > 11 cells, 3 mice). (f) Example traces of the first AP fired at rheobase in a 12‐month‐old mouse and a close‐up of APs showing the position of the AP threshold (green cross) (*n* > 9 cells, 3 mice). Averaged data are presented as mean ± standard error of the mean.

To further investigate mechanisms whereby the pSTR is restored in older mice, we conducted additional whole‐cell patch clamping experiments in isolated retinal flatmounts whereby action potential (AP) firing was generated in response to current injection (Figure 4a,d). In 3‐month old mice, input–output curves of AP firing in response to increasing current was not significantly altered at 3‐ and 7‐days post injury compared to control values (Figure 4b). AP threshold (the current level at which an AP is first propagated) was also unchanged following injury (Figure 4c). Similar to young mice, AP firing was unchanged after injury in 12‐month‐old mice (Figure 4e), although we did notice that AP firing was overall marginally increased in 12‐month old controls compared to 3‐month old controls. These results are consistent with our VEP data and suggest that RGC output is unaltered following IOP injury in both young and older mice. Unlike in young mice, AP threshold in older 12‐month‐old mice was significantly hyperpolarised at 7‐days (*p* < 0.05) and 28‐days (*p* < 0.05) after IOP elevation compared to controls (Figure 4f), suggesting that while RGC synaptic inputs remain decreased following injury, output is maintained by modulating AP threshold. No other changes in AP kinetics were observed in response to injury in either young or older mice (Table 1).

**TABLE 1:** Action potential (AP) kinetics of patch‐clamped RGCs in 3‐ and 12‐month‐old mice following IOP injury.

3‐month‐old	Control (*n* = 13)	Day 3 (*n* = 10)	Day 7 (*n* = 15)	*p*
AP amplitude (mV)	66.92 ± 3.8	68.61 ± 4.4	77.57 ± 2.6	0.067
AP rise‐time (ms)	0.64 ± 0.07	0.65 ± 0.05	0.61 ± 0.10	0.942
Rheobase (pA)	29.23 ± 3.6	24.00 ± 2.7	32.67 ± 4.6	0.357
Afterhyperpolarisation amplitude (mV)	3.70 ± 1.8	3.38 ± 1.8	1.53 ± 1.8	0.635
Input resistance (MΩ)	500.7 ± 61.7	569.8 ± 91.2	433.0 ± 58.9	0.392

12‐month‐old	Control (*n* = 11)	Day 7 (*n* = 14)	Day 28 (*n* = 12)	*p*
AP amplitude (mV)	61.18 ± 3.7	71.02 ± 3.8	69.56 ± 2.5	0.122
AP rise‐time (ms)	0.63 ± 0.08	0.48 ± 0.03	0.61 ± 0.08	0.217
Rheobase (pA)	23.64 ± 2.4	27.14 ± 3.4	20.00 ± 0.01	0.145
Afterhyperpolarisation amplitude (mV)	0.93 ± 2.8	1.88 ± 2.2	1.43 ± 1.6	0.954
Input resistance (MΩ)	557.2 ± 73.5	441.2 ± 83.1	683.2 ± 114.7	0.182

*Note:* Mean ± standard error of the mean; one‐way ANOVA with Tukey–Kramer post hoc test.

### 2.6 Acute Intraocular Pressure Injury Causes a Persistent Loss of Retinal Ganglion Cell Synapses in Young and Middle‐Aged Mice

**FIGURE 5:**
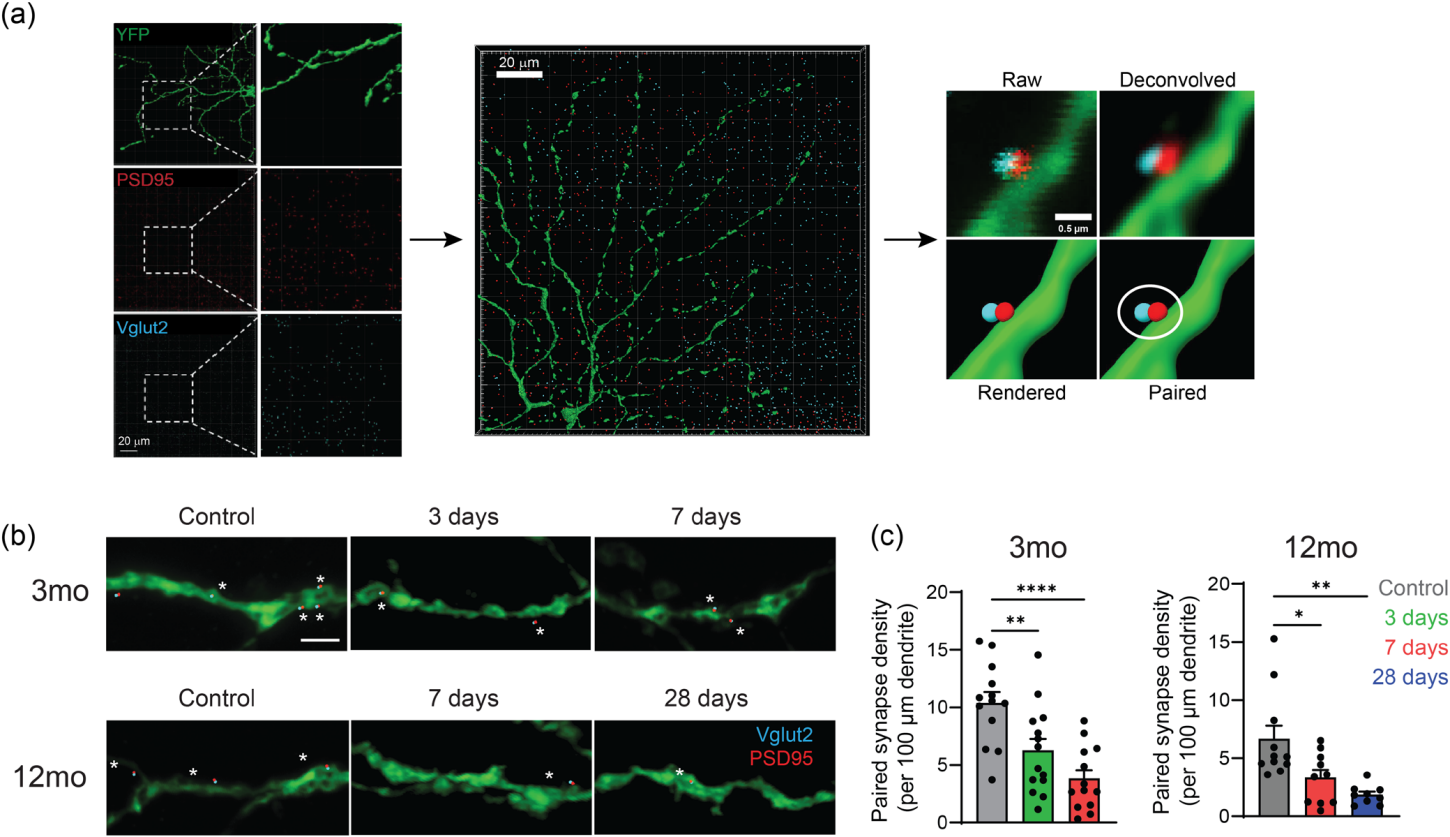
Acute intraocular pressure (IOP) injury causes a persistent loss of retinal ganglion cell (RGC) synapses in young and middle‐aged mice. (a) Example photomicrographs of YFP labeled RGC dendrites (green), PSD95 labeled postsynapses (red) and Vglut2 labeled presynapses (cyan). Using Imaris image analysis software, RGC dendrites were rendered as a “surface” and pre‐ and postsynapses were rendered as “spots”. Following Nyquist sampling and deconvolution to achieve high spatial resolution, postsynapses along the dendrite were paired with a presynaptic partner. (b) Example images of paired synapses within 50 nm of RGC dendrites in 3‐month‐old and 12‐month‐old mice. Scale bar = 3 μm. (c) Quantification of paired synapses in mice after IOP elevation. Paired synapses were defined as postsynapses within 50 nm of RGC dendrites and within 50 nm of a presynapse, normalized to dendrite length (*n* > 13 cells, 7 mice). *p < 0.05, **p < 0.01, ****p < 0.0001; one‐way ANOVA with Tukey–Kramer post hoc test. Averaged data are presented as mean ± standard error of the mean.

After IOP elevation, RGC dysfunction in 12‐month old mice persists much longer than in 3‐month old mice. As age causes a loss of synaptic density in the inner plexiform layer of the retina (reviewed in Petralia, Mattson, and Yao 2014), we hypothesized that age‐related changes in synaptic connectivity may affect the rate of RGC recovery following IOP injury. To visualize excitatory synapses, we immunolabeled RGC dendrites with YFP and co‐stained excitatory presynapses with Vglut2 and excitatory postsynapses with PSD95 (Figure 5a,b). Tile images of a whole RGC dendritic tree were acquired and high spatial resolution was achieved by Nyquist sampling and deconvolution. As postsynapses do not always have a presynaptic partner, we quantified paired synapses as a more physiologically relevant measure of function (Stevens et al. 2007). Using Imaris software of a 3D image, RGC dendrites were rendered as a surface and immunolabeled synapses were rendered as “spots” in order to pair excitatory postsynapses with a presynaptic partner. Paired synapses, which were defined as postsynapses within 50 nm of a presynapse and within 50 nm to the dendritic tree, were then quantified across the entire area of a RGC dendritic tree and normalized to dendrite length. In 3‐month‐old mice, the density of paired synapses was significantly decreased at 3‐days (*p* < 0.01) and 7‐days (*p* < 0.0001) after IOP elevation compared to uninjured controls (Figure 5c). In 12‐month‐old mice, the density of paired synapses was also significantly decreased at 7‐days (*p* < 0.05) and 28‐days (*p* < 0.01) after IOP elevation (Figure 5c). Data from uninjured control eyes showed an age‐related reduction in the number of paired excitatory synapses; 10.4 ± 3.78 paired synaptic puncta in 3‐month‐old mice versus 6.7 ± 3.78 paired synaptic puncta in 12‐month‐old mice. These data suggest that functional recovery observed by ERG is not consequent to a recovery in excitatory synapse number.

### 2.7 Acute Intraocular Pressure Injury Reduces Retinal Ganglion Cell Dendritic Complexity and Causes Changes in Axon Initial Segment Length in Middle‐Aged Mice

**FIGURE 6:**
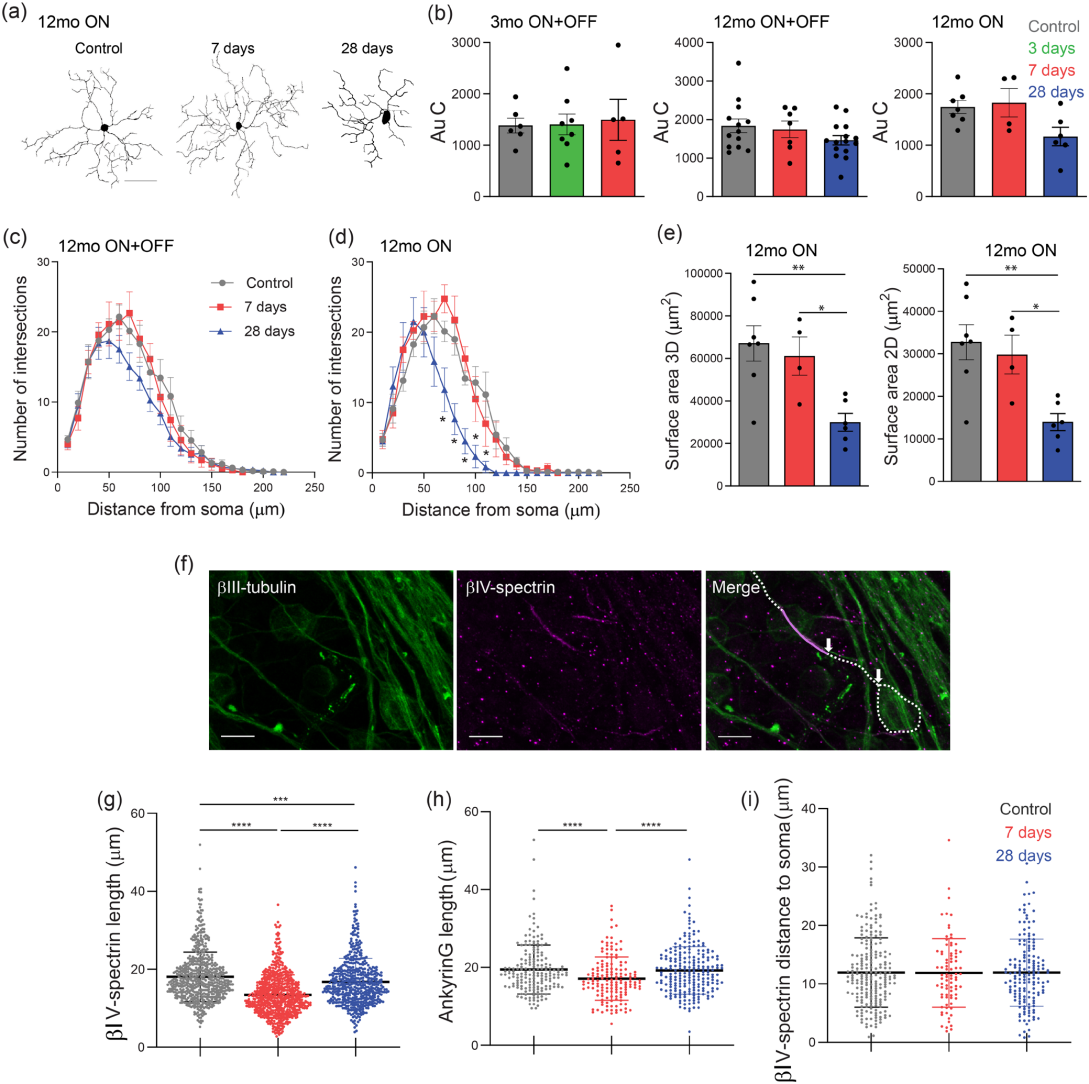
Acute intraocular pressure (IOP) injury causes a loss of retinal ganglion cell (RGC) dendritic complexity and changes to the length of the axon initial segment (AIS) in older mice. (a) Flattened 2D tracings of Biocytin‐filled RGCs were used for dendritic morphological analysis. Scale bar = 50 μm. (b) Overall dendritic complexity was measured by area under the Sholl curve (AuC). [(3‐month ON + OFF: Control *n* = 6; 7‐days *n* = 8; 28‐days *n* = 5), (12‐month ON + OFF: Control *n* = 13; 7‐days *n* = 7; 28‐days *n* = 15), (12‐month ON: Control *n* = 7; 7‐days *n* = 4; 28‐days *n* = 6)]. (c) Sholl profiles from all RGCs and (d) ON‐RGCs showing the number of dendritic intersections relative to distance from soma in 12‐month‐old mice after IOP injury. [(12‐month ON+OFF: Control *n* = 13; 7‐days *n* = 7; 28‐days *n* = 15), (12‐month ON: Control *n* = 7; 7‐days *n* = 4; 28‐days *n* = 6)]. *p < 0.05; two‐way ANOVA with Tukey's post hoc test. (e) Dendritic arbour area of ON‐RGCs in 12‐month‐old mice was measured by convex hull of the dendritic field in both two and three dimensions. *p < 0.05, **p < 0.01; one‐way ANOVA with Tukey–Kramer post hoc test. (f) Maximum intensity projections demonstrating immunostaining of the AIS with βIV‐spectrin (purple), the RGC soma and axon with βIII‐tubulin (green), and overlaid (merge). Measurement of AIS length (solid magenta line) and the RGC soma and axon (dotted line) are demonstrated. Arrows demonstrate the distance between proximal end of the AIS and the soma. (g) Quantification of βIV‐spectrin length in response to IOP elevation in 12‐month‐old mice (Control *n* = 4 mice, [604 cells]; 7‐days *n* = 6, [734]; 28‐days *n* = 7, [549]). (h) Quantification of AnkyrinG length in response to IOP elevation in 12‐month‐old mice (Control *n* = 2 mice [185 cells]; 7‐days *n* = 3 [154]; 28‐days *n* = 3 [224]). (i) AIS position with respect to the soma following injury (Control *n* = 4 mice [211 cells]; 7‐days *n* = 3 [83]; 28‐days *n* = 5 [160]). ***p < 0.001, ****p < 0.0001; Kruskal‐Wallis ANOVA with Dunn's post hoc test. Averaged data are presented as mean ± standard error of the mean.

To further assess the increase in intrinsic excitability observed in older mice with ERG recovery (shown earlier in Figure 4), we investigated structural changes to the dendritic arbour and axon initial segment (AIS) of RGCs. In models of chronic IOP elevation, changes in RGC dendrite morphology have been described prior to extensive cell death (Berry et al. 2015). To assess cell morphology after IOP injury, Biocytin‐filled RGCs were traced using Neurolucida software, and a Sholl analysis and various dendritic parameters were extracted (Figure 6a). We have previously shown that there is no significant difference in RGC morphology or Sholl profiles with age or at 7‐days after IOP elevation in 3‐ and 12‐month‐old mice (Lee et al. 2022). These findings were confirmed in our current experiments (Figure 6b) and we then expanded our analysis by investigating RGC morphology from 12‐month‐old mice in a recovered state (i.e., 28‐days post‐injury). Taking all RGCs together (ON‐ and OFF‐cells), no significant differences were detected in multiple parameters of dendritic morphology at either 7‐days or 28‐days post‐injury in 12‐month‐old mice compared to control values (Table 2). However, 28‐day values for dendritic length, dendritic field surface area, Sholl intersections and area under the curve had lower means than control values. This is seen as a leftward shift and reduction in height of the Sholl curve (Figure 6c), indicating a minor decrease in arbour complexity in response to injury. As ON‐ and OFF‐RGCs may be preferentially susceptible to IOP‐induced damage, we next performed a differential assessment of RGC subtypes. We found a significant reduction in the number of Sholl intersections from ON‐RGCs at all points between 60 μm and 110 μm from the soma at 28‐days compared to baseline (Figure 6d). Furthermore, ON‐RGCs exhibited a significant 57% reduction in two‐dimensional field area at 28‐days compared to baseline (*p* < 0.01) and a similar 55% reduction in three‐dimensional area (*p* < 0.01) (Table 2, Figure 6e). Within the OFF‐RGC group, no significant differences were seen (data not shown). Our analysis suggests that ERG recovery is not consequent to re‐extension of dendrites in older mice.

**TABLE 2:** Dendritic arbour parameters of RGCs in 12‐month‐old mice following IOP injury.

ON + OFF cells	Control (*n* = 13)	Day 7 (*n* = 7)	Day 28 (*n* = 15)	*p*
Number of Sholl intersections (*n*)	185.8 ± 18.4	176.6 ± 21.5	148.3 ± 12.1	0.21
Sholl AUC	1835 ± 181	1746 ± 214	1463 ± 120	0.21
Soma area (μm^2^)	103.7 ± 7.8	119.9 ± 14.3	95.57 ± 8.72	0.28
Dendritic length (μm)	2772 ± 266	2641 ± 280	2343 ± 183	0.38
Dendrite surface area (μm^2^)	4605 ± 428	4154 ± 530	3873 ± 333	0.40
Dendritic field surface area (3D) (μm^2^)	67,767 ± 8468	55,785 ± 5989	55,861 ± 9330	0.55
Dendritic field surface area (2D) (μm^2^)	32,727 ± 4249	26,848 ± 3141	26,945 ± 4592	0.57
Max branch order (*n*)	13.23 ± 1.42	15.00 ± 2.07	12.53 ± 0.83	0.49
Number of segments (*n*)	159.8 ± 24.0	166.4 ± 16.0	146.7 ± 16.0	0.79

ON cells	Control (*n* = 7)	Day 7 (*n* = 4)	Day 28 (*n* = 6)	*p*
Number of Sholl intersections (*n*)	176.9 ± 13.16	185.3 ± 28.24	119.2 ± 18.31	0.049
Sholl AUC	1745 ± 128	1829 ± 278	1169 ± 181	0.045
Soma area (μm^2^)	104.3 ± 12.8	130.3 ± 24.5	86.5 ± 8.5	0.18
Dendritic length (μm)	2665 ± 216	2727 ± 354	1915 ± 266.6	0.09
Dendrite surface area (μm^2^)	4276 ± 408	4084 ± 568	2946 ± 365	0.09
Dendritic field surface area (3D) (μm^2^)	67,084 ± 8312	61,147 ± 8995	29,956 ± 4159	**0.0055** **
Dendritic field surface area (2D) (μm^2^)	32,754 ± 4104	29,830 ± 4559	13,961 ± 4592	**0.0046** **
Max branch order (*n*)	13.00 ± 2.09	14.00 ± 3.39	12.33 ± 0.56	0.87
Number of segments (*n*)	175.1 ± 38.10	158.8 ± 15.61	160.5 ± 23.69	0.92

*Note:* Mean ± standard error of the mean; one‐way ANOVA with Tukey–Kramer post hoc test.

** *p* < 0.01.

The AIS is responsible for initiating and shaping the AP in neurons. Shortening of the AIS and movement away from the soma are associated with reduced intrinsic excitability, which is a mechanism by which neurons reduce sodium conductance and intrinsic excitability (Kaphzan et al. 2011; Schafer et al. 2009). To quantify AIS length in RGCs, retinal flatmounts from 12‐month‐old mice were labeled with an antibody against βIV‐spectrin (Figure 6f). We found that βIV‐spectrin length decreased by 26% (*p* < 0.0001) at 7‐days post injury and then recovered to 93% (*p* < 0.001) of control length by 28‐days (Figure 6g). To validate these changes, antibodies against AnkyrinG were used to label AIS in a second cohort of mice. Results confirmed a similar pattern of reversible AIS shortening in 12‐month‐old mice in response to IOP injury (Figure 6h). The change in AIS length in 12‐month‐old mice was not associated with a change in proximity to soma (Figure 6i). These data show that AIS length correlates with loss and recovery of the RGC ERG signal after IOP injury.

## 3 Discussion

This study has provided new insights into synaptic changes in the mouse inner retina in response to advancing age and IOP injury. We report a gradual and significant loss of function across all major types of retinal neurons with normal aging in the C57BL/6J mouse, despite minimal cell loss. In response to an acute IOP injury, we see RGC function diminish and recover with no or minimal cell loss, suggesting that alterations in intrinsic neuronal excitability or cellular morphology underlie retinal dysfunction. In support of this theory, we saw that RGCs can modulate excitatory and inhibitory synapse function, AP threshold, synaptic density, and AIS length after IOP injury, and that these responses to injury differ with mouse age. We show that young mice regain their excitatory inputs and decrease their inhibitory inputs to recover RGC excitability. In contrast, older mice do not regain excitatory inputs but instead become intrinsically more excitable by modulating AP thresholds and AIS length. We further show that the density of paired synapses is significantly decreased in young and older mice in response to injury and that there are minor alterations in dendritic complexity in older mice only.

Using the electroretinogram to assess retinal cell function, others have reported a decline in function with increasing age in the C57BL/6 mouse retina that is similar to our current data (Ferdous et al. 2021; Gresh et al. 2003; Samuel et al. 2011). An age‐related decline in retinal function has also been reported in humans (Birch and Anderson 1992). Optical factors such as cataract, other pre‐retinal media changes, or changes in ocular impedance do not appear to explain this age‐related ERG amplitude loss (Park et al. 2023). While previous work has focussed predominantly on the photoreceptor system of rods and cones and bipolar cells, here we provide new information specific to the inner retina by capturing and analyzing pSTR amplitudes, which are a measure of the local field potentials derived from RGCs. Whilst we detect a gradual and significant age‐related loss of function across all major retinal cell types, we show that the rate of RGC impairment beyond 12‐months of age is greater than can be accounted for by the loss of upstream retinal signaling, suggesting that RGCs are more susceptible to the negative impacts of aging than photoreceptors.

Previous reports of photoreceptor survival with age in the mouse retina are inconsistent; some report cell loss (Ferdous et al. 2021) while others do not (Samuel et al. 2011). While most agree that the number of RGC somas remain constant across age in mammals, the RGC axon itself is highly vulnerable to degeneration with increasing age (reviewed in Calkins 2013). In this study, we found that numbers of inner, mid and outer retinal neurons remained stable throughout the mouse lifespan although we cannot rule out small losses. We did not investigate other structural changes such as retinal volume, cell shape, or the number and density of RGC axons, all of which have previously been shown to change with age in the mouse (Cepurna et al. 2005; Harwerth, Wheat, and Rangaswamy 2008; Samuel et al. 2011). Nonetheless our results suggest that the loss in retinal function with age is not caused by a substantial reduction in cell number in the C57BL/6J mouse.

Using a time‐defined IOP injury model, we confirm prior literature showing RGC functional recovery is substantially delayed in older mice (Chrysostomou et al. 2024; Lee et al. 2022). The loss and recovery of RGC‐derived ERG signals in response to injury cannot entirely be explained by cell loss as we saw no significant reduction in cell number in young or older mice. It is still possible that some of the changes in ERG waveforms after injury are consequent to loss of a smaller proportion of RGCs in older mice that do not reach statistical significance with the number of retina that were counted. It is also possible that slower functional recovery in older mice is consequent to underlying damage in remaining RGCs; previous studies show that a large population of surviving Brn3a‐positive RGCs lose their capacity for active retrograde axonal transport (Vidal‐Sanz et al. 2012). Nonetheless, RGC function was substantially impacted by aging and IOP elevation as both patch‐clamp and ERG recordings were altered and reflected changes in the residual, still viable RGCs, in response to current and light respectively.

Our findings using whole‐cell patch‐clamp electrophysiology has provided new insights into the synaptic mechanisms that contribute to RGC dysfunction and subsequent recovery after IOP injury. The ability of young mice to increase their eEPSC amplitude and decrease their eIPSC amplitude appears to compensate for the loss of excitatory synapses after injury and allows RGCs to maintain their electrical output. This is consistent with the concept of homeostatic plasticity in which neuronal responses to changes in network activity act in the opposite direction to restore normal activity levels. Synaptic scaling or a global increase in excitatory synapse strength by modulation of AMPA receptors may underlie this plasticity (Gainey et al. 2009; Hanes et al. 2020). Unlike young mice, older mice appear unable to compensate for the loss of synapses after injury by increasing their eEPSC amplitude or altering their eIPSC amplitude. One reason why we may not see a change in inhibitory synapse function is that eIPSC amplitude is already reduced in 12‐month old mice compared to 3‐month old mice and potentially cannot be reduced further following IOP elevation. We also observed an age‐related reduction in the size of ERG oscillatory potentials, which are thought to reflect inhibitory feedback pathways initiated by amacrine cells. It should be borne in mind that the proposed mechanistic differences in the way that RGCs in young and old retina regain function were derived from experiments where IPSCs and EPSCs were induced by current, not by light stimulation. As such, further work is required to verify whether similar changes are observed in response to incident light. Concurrent pharmacologic interventions would add further validation to these initial findings.

Rather than altering eEPSC or eIPSC amplitudes, it appears that older mice compensate for synapse loss by altering their AP threshold and becoming intrinsically more excitable. Interestingly, even under control conditions we saw that AP firing was slightly increased in older mice. Our recent observations that 12‐month‐old mice are unable to recover from a second IOP challenge (Chrysostomou et al. 2024) suggests that restoration of ERG amplitudes through alteration in AP threshold may be a transient compensation rather than true recovery as it renders RGCs more vulnerable to a second injury. In contrast, younger mice are able to fully recover from a similar second IOP challenge, suggesting that restoration of excitatory inputs does not render RGCs vulnerable to degeneration after a second injury. Many of the differences in AP counts observed in the current study were not statistically significant. The “fall off” after reaching maximum AP number is a well‐recognized phenomenon that can also occur also in brain slices. A reduction in AP number at currents above Vmax is well recognized but hard to interpret the underlying significance or cause. Changes at currents above Vmax generally reflect a loss of excitability that could be due to changes in channel dynamics (inactivation, adaptation), changes in ion gradients, or technical artifacts.

We demonstrated non‐recoverable loss of excitatory paired synapses in 3‐ and 12‐month old mice after acute IOP elevation. This in itself is not novel as previous studies have shown that excitatory synapses are decreased in models of IOP elevation (Agostinone and Di Polo 2015; Park, Kim, and Park 2014; Van Hook 2022), and synaptic disassembly is considered an early hallmark of neurodegenerative diseases in general (Kulkarni et al. 2022; Meftah and Gan 2023). The reduction of paired synapses in our experiments occurred in the absence of significant RGC loss or loss of optic nerve output, supporting that changes in excitatory input are an early response to injury. Our observation of a lower number of paired synapses in 12‐month‐old mice compared to 3‐month old mice is consistent with previous reports showing a general reduction of inner retinal synapse number with normal aging in the C57BL/6 mouse (Samuel et al. 2011). We have extended previous literature by correlating synaptic changes with RGC‐derived ERG signals after IOP injury in both young and older mice. While the loss of excitatory synaptic input correlates with a reduction of inner retinal function in both 3‐ and 12‐month‐old mice, the persistent reduction in paired synapses does not explain how the pSTR amplitude is recovered at either age. One mechanism that could explain the recovery of RGC function is a change in the distribution or localisation of paired synapses, which is known to alter neuronal excitability and has been reported in neurodegenerative diseases (Halpain, Spencer, and Graber 2005). A further mechanism could be compensation for the loss of paired synapses by an increase in presynaptic glutamate release (Johnson and Kerschensteiner 2014). We are actively pursuing these topics.

We found evidence of changes in RGC morphology after IOP injury in 12‐month‐old mice, as seen by a reduction in the number of Sholl intersections and reductions in dendritic complexity. These changes were most prominent in ON‐RGCs and are consistent with our previous report using the same acute IOP injury model (Lee et al. 2022). Overall, changes in RGC dendritic field areas and complexity in response to IOP elevation are unclear: some studies report significant changes (Ou et al. 2016; Risner et al. 2018) while others report no change (Della Santina et al. 2013; El‐Danaf and Huberman 2015). These discrepancies may be due to inherent differences between the response of ON‐ versus OFF‐RGCs to IOP injury and differences between responses to chronic vs. acute IOP elevation.

We provide evidence of reversible structural plasticity of the RGC AIS in response to elevated IOP, which correlates with ERG data showing loss and recovery of the RGC‐derived pSTR. This suggests that changes to AIS length may underlie the alterations in ERG signaling in this injury model as the AIS is the location for AP generation and is thus a key determinant of neuronal excitability (Kole and Stuart 2012). It is plausible to hypothesize that an injured RGC may shorten its AIS as an adaptive mechanism to reduce excitability and frequency of AP firing or to change threshold voltage as a neuroprotective mechanism. The reduced metabolic demands of AP firing may serve as a means of conserving energy for cellular repair processes after injury. In support of this hypothesis, prior studies have demonstrated structural changes to the AIS such as lengthening, shortening, and distal or proximal location shifts in response to brain injury or disease (Hinman, Rasband, and Carmichael 2013; Kaphzan et al. 2011). However, none of these studies demonstrate reversibility of these changes like those we describe here after IOP elevation.

## 4 Conclusions

We have demonstrated that advancing age and IOP elevation cause a reduction in the amplitude of full‐field ERG signals with minimal neuronal loss. We show that the reduction in the inner retinal‐derived pSTR signal with aging and injury is likely caused by a loss of RGC excitatory synaptic input, specifically a loss of paired excitatory synapses and a reduction in eEPSC amplitude in young and middle‐aged mice. While young mice modulate their synaptic input to recover RGC function following injury, older mice compensate by altering their AP threshold to increase intrinsic excitability and this is associated with an increase in AIS length. As IOP elevation does not appear to cause substantial changes in axonal conduction measured by the VEP or AP firing, our findings suggest that RGCs are able to restore function in the absence of reconstituting new synapses. Our data support the hypothesis that age‐related functional loss and early stages of retinal dysfunction following injury are driven by changes in RGC synaptic connectivity, rather than major alterations in cell number.

## 5 Materials and Methods

### 5.1 Experimental Animals

All experimental procedures conformed to the ARVO Statement for the Use of Animals in Ophthalmic and Vision Research under the guidelines of the NHMRC Code of Practice for the Care and Use of Animals for Experimental Purposes in Australia. Procedures were approved by the Alfred Medical Research & Education Precinct Animal Ethics Committee and The Florey Animal Ethics Committee (18‐112‐FINMH). C57BL/6J mice and B6.Cg‐Tg (Thy1‐YFP)HJrs/J mice (Stock No: 003783; The Jackson Laboratory, USA) were maintained on a 12‐h light/dark cycle and housed in a PC2 facility with ad libitum access to food and water. Male and female mice were used equally throughout the study. Genotyping confirmed that experimental mice were homozygous for the Nnt mutation and did not carry the rd8 mutation.

### 5.2 Electroretinography

The full‐field flash ERG was recorded using an Espion Diagnosys system as described previously (Crowston et al. 2015). Retinal responses to a series of stimulus intensities (1.3e−6 to 100 cd.s/m^2^) were recorded simultaneously from both eyes. The RGC‐mediated positive scotopic threshold response (pSTR) is elicited with low intensity illumination and is derived predominantly from RGCs in the rodent retina (Saszik, Robson, and Frishman 2002). Amplitudes of the pSTR were measured at a fixed time of 110 ms after a flash stimulus of 6.3e−6 cd.s/m^2^, which coincides with the pSTR peak. Amplitudes of waveforms derived from photoreceptors (a‐wave) and ON‐bipolar cells (b‐wave) were measured in response to a series of flash stimuli from 0.003 to 100 cd.s/m^2^. In a subset of mice, full‐field flash visual‐evoked potentials (VEPs) were measured following implantation of cranial screw electrodes. Mice were anesthetized using a mixture of ketamine (60 mg/kg) and xylazine (10 mg/kg) and then secured in a stereotaxic frame (RWD Life Sciences, China). A 5 mm^2^ area of skin overlying the scalp was excised to expose the skull and pilot holes were drilled for electrode placement (0.1 mm diameter, 2 mm length). Electrodes were placed according to previously published protocols: the active electrode was placed over the primary visual cortex 3.6 mm caudal and 2.3 mm lateral to the bregma and the inactive electrode was placed 1 mm rostral and 1 mm lateral to the bregma. A subcutaneous needle (ADInstruments, Australia) reference electrode was placed at the base of the tail. Electrodes were secured in place with dental cement (Dentsply, Germany). Electrode implantation was performed a minimum of 2 days prior to recordings to enable complete recovery. VEPs were recorded and averaged following stimulation with 20 flashes (1.29 log cd.s/m^2^), with a 20 ms inter‐stimulus interval. VEP amplitudes and latencies were calculated at the first positive and first negative trough (N1).

### 5.3 Acute Intraocular Pressure Elevation

Injury to RGCs was induced by short‐term elevation of IOP, a well‐characterized non‐ischemic insult that has been described in detail elsewhere (Crowston et al. 2015). The anterior chamber of the mouse eye was cannulated with a 50 μm pulled borosilicate needle connected via polyethylene tubing to a syringe mounted on a motorized pump (PHD Ultra CP; Harvard Apparatus, Massachusetts, USA) and a pressure transducer (Transpac IV, Abbott Critical Care Systems, Sligo, Ireland). The syringe and tubing were filled with sterile‐filtered endotoxin‐tested Hanks' balanced salt solution (HBSS, JRH Biosciences, Lenexa, KA, USA). The pump was calibrated to maintain a constant IOP within 1 mmHg of the target. Resting IOP was measured by the PHD Ultra CP system immediately after cannulation of the anterior chamber, before IOP was elevated to 50 mmHg for 30 min, and was monitored continuously during the IOP elevation phase. Measures of IOP were further confirmed by rebound tonometry (Icare Tonovet).

### 5.4 Immunohistochemistry of Retinal Sections and Cell Quantification

Eyes were enucleated and immersion‐fixed in 4% paraformaldehyde for 3 h, followed by overnight cryoprotection in 15% sucrose. Eyes were embedded in optimal cutting temperature medium and 12 μm sections were cut through the pupillary‐optic nerve axis. Cryosections were stained with Hoechst (1:10,000) using standard procedures (Kezic et al. 2013). Retinal thickness measurements were made on digital images of Hoechst‐stained cryosections taken with an AxioCam Carl Zeiss microscope. Sections were scanned from the superior to inferior edge and the retinal thickness was determined every 500 μm (a total of eight measurements per retina). At each measurement location, the thickness of the outer nuclear layer (ONL) and the thickness of the retina, from inner to outer limiting membrane (ILM–OLM), were recorded. The ratio of the thickness of the ONL to the thickness of the retina (measured from the ILM to the OLM) was used for analysis, to account for obliquely cut sections. The average thickness across the 8 fields was calculated for each retina and used for statistical analysis.

### 5.5 Immmunohistochemistry of Retinal Flatmounts

Retinas were dissected from freshly enucleated eyes, flatmounted under a dissecting microscope and immersion‐fixed in 4% paraformaldehyde for 1 h. Retina flatmounts were washed in 1× PBS for 30 min, incubated in blocking buffer (1% goat serum and 0.2% triton X100, 0.05% ProClin in 0.1 M phosphate buffer) at room temperature for 1 h before incubation with primary antibodies against Brn3a (Merck Pte Ltd., 1:500), Vglut2 (Merck, 1:1000), PSD95 (Abcam, 1:500) and YFP (Abcam, 1:2000) at room temperature overnight. Flatmounts were then washed in 1× PBS three times (10 min each time), incubated with secondary Alexa Fluor antibodies (405, 488 or 595; Abcam, 1:2000) for 1 h before placing coverslips with mounting medium (Vectashield; Vector Lab, Burlingame, CA).

#### 5.5.1 RGC Quantification

To assess RGC density, retinal flatmounts were divided into 4 quadrants: superior, temporal, nasal, and inferior. Retinal micrographs were taken at 20× magnification in 4 standard sampling fields (0.56 × 0.42 mm) from the centre of each quadrant using an AxioCam Carl Zeiss microscope. Brn3a immunoreactive cells from the 4 fields per retinal sample, covering a total area of 0.95 mm^2^, were quantified using ImageJ software. The average density per mm^2^ across the 4 fields was calculated and used for statistical analysis.

#### 5.5.2 Synapse Quantification

Retinal flatmounts were imaged using a Zeiss 780 confocal microscope with a 63× oil objective (1.4 NA) with a voxel size of 0.035 × 0.035 × 0.107 μm^3^. Images were deconvolved using Huygens deconvolution software and analyzed using Imaris Image Analysis Software (Oxford Instruments, UK). RGC dendrites were rendered as a surface and pre‐ and post‐synapses were rendered as ‘spots’ with a spot size of 0.25 μm. Using the ‘find spots close to surface’ function, presynapses and postsynapses within 0.5 μm of the dendrite were quantified. Using the ‘find spots close to spots’ function, postsynapses within 0.5 μm of the dendrite and within 0.5 μm of a presynapse were quantified.

### 5.6 Flatmount Preparation for Electrophysiology

Animals were anesthetized with 2% isoflurane and cervically dislocated. The eye was quickly enucleated, the cornea pierced with a 19 _G_ needle and placed into a cold dissecting solution consisting of (mM): 125 Choline‐Cl, 2.5 KCl, 0.4 CaCl_2_, 6 MgCl_2_, 1.25 NaH_2_PO_4_, 26 NaHCO_3_, 20 D‐glucose saturated with 95% O_2_ plus 5% CO_2_. The retina was then dissected, vitreous removed and hemisected. The retina was then incubated at room temperature for 1 h in Ames medium (Sigma Aldrich; NSW, AUS) solution saturated with 95% O_2_ and 5% CO_2_ (carbogen gas) before electrophysiology was performed.

### 5.7 Whole‐Cell Patch‐Clamp Electrophysiology

Retinal hemi‐sections were transferred to a recording chamber with the ganglion cell layer facing up and set in place with a harp. The recording chamber was placed on an upright microscope (Slicescope Pro 1000; Scientifica, UK) and perfused with AMES medium (AMES medium, 22.6 mM NaHCO_3_) saturated with carbogen gas at room temperature and at a rate of 2 mL/min. RGCs and bipolar cells were identified visually using a fluorescence lamp under a 40× water‐immersion objective (Olympus, Japan) and a CCD camera (IEEE 1394; FOculus, Germany).

#### 5.7.1 Stimulating Electrode

Glass electrodes (BF150‐110‐10; Sutter instruments, USA) were pulled using a Flaming Brown micropipette puller (Model P‐1000, Sutter Instruments, USA) to produce a resistance of 0.5–1 MΩ. Silver wire was wrapped around the glass electrode and connected via insulated wire to a stimulating box. Glass electrodes were painted in silver‐chloride paint, filled with Ames media (AMES medium, 22.6 mM NaHCO_3_) and fastened into a microelectrode holder containing a silver chloride coated electrode. Positive pressure was added to the stimulating electrode and the retinal inner limiting membrane was pierced and the stimulating electrode placed within the bipolar cell layer.

#### 5.7.2 Recording Electrodes

Recording electrodes (BF150‐86‐7.7HP; Sutter instruments, USA) were pulled using a Flaming Brown micropipette puller (Model P‐1000, Sutter Instruments, USA) to produce a resistance of 4–7 MΩ and filled with internal solution. Evoked synaptic currents were recorded with electrodes containing (mM): 135 CdMeSO_4_, 8 NaCl, 10 HEPES, 2 Mg_2_ATP, 0.3 Na_3_GTP, 7 Phosphocreatine, 10 EGTA (pH 7.3). Action potentials were recorded with electrodes containing (mM): 125 K‐gluconate, 10 photphocreatine, 5 BCL, 2 MgCl_2_.6H_2_O, 10 HEPES, 0.1 EGTA, 4 MgATP, 0.3 NaGTP. Electrodes were fastened in a microelectrode holder containing a silver chloride coated electrode and positive pressure was added. The retinal inner limiting membrane was pierced and RGCs were patched within the ganglion cell layer, immediately above the stimulating electrode.

#### 5.7.3 Evoked postsynaptic recordings

Patch‐clamp recordings were made in current clamp mode using PatchStar micromanipulators (Scientifica, UK) and an Acon Multiclamp 700B patch‐clamp amplifier (Molecular Devices, USA). Data were acquired using pClamp software (v10; Molecule Devices, USA) with a sampling rate of 50 kHz and low pass Bessel filtered at 10 kHz (Digidata, 1440a; Axon). EPSCs were evoked using a single 0.5 mA, 1 ms pulse repeated 10 times every 10 s (Digital Isolator, Model BIN8‐9 V; Getting Instruments, USA). Excitatory postsynaptic currents (EPSCs) were recorded by holding the RGCs at −70 mV and inhibitory postsynaptic currents (IPSCs) were recorded by holding the RGCs at +10 mV.

### 5.8 Patch‐Clamp Data Analysis

Recordings were analyzed using AxoGraph software (AxoGraph 1.7.6, USA). Cells with an access resistance of < 40 MΩ were analyzed. All 10 recordings had their baseline subtracted from 0 to 250 ms. The peak evoked EPSC (eEPSC) amplitude was detected from 500 to 700 ms and the peak shape was analyzed to determine eEPSC onset, rise time and decay time. Spontaneous EPSC events were identified using a detection threshold set at two times the standard deviation of background noise. Each event was manually confirmed. EPSC amplitude and frequency were calculated and cumulate probability curves constructed. No series resistance was applied. Individual action potentials (APs) were identified using an amplitude threshold (50 mV relative to pre‐event baseline). All AP kinetics were calculated for the first AP generated following injection of a rheobase current. AP amplitude was calculated relative to pre‐event baseline. AP threshold was determined at the membrane potential where voltage acceleration reached 10 V/s. AP rise‐time was calculated as the period between 20% and 80% of maximal AP amplitude. Afterhyperpolarisation amplitude was calculated relative to AP threshold. The input resistance was calculated from the voltage deflection relative to baseline that occurred from injection of a −60 pA, 400 ms duration test pulse.

### 5.9 Single Cell Filling and Imaging

Following whole cell patch clamping and electrophysiological recording, individual RGCs were filled with extracellular solution, biocytin (0.5%, Sigma‐Aldrich) and Alexa Hydrazide 488 (Thermofisher Scientific, VIC, Australia). Three‐dimensional tiled image stacks were created on a Zeiss LSM‐780 using a Plan‐Apochromat 63X/1.4 NA Oil DIC objective (Zeiss, Germany) 1.5× magnification and frame size 1024 × 1024 at resolution satisfying Nyquist sampling (XY resolution 0.088 nm, Z‐step interval 0.400 nm). Stacks were imaged from the retinal nerve fiber layer (RNFL) to the inner nuclear layer, using a tile scan stitched with the ZEN2 software (Zeiss, Germany) to encompass the entire RGC dendritic tree. Complete dendritic morphological reconstructions were made with Neurolucida software (11.06.2, MBF Bioscience, VT, USA) using manual and interactive tracing. Sholl analysis was conducted by calculating the number of dendritic intersections with concentric three‐dimensional spheres extended from the soma at 10 μm intervals (Morgan et al. 2006). Total dendritic length was calculated as the length of all branches of the dendritic tree. RGCs were classified as ON‐ or OFF‐cells based on depth of dendritic stratification in the inner plexiform layer (Coombs et al. 2006) and sampling was subsequently performed across equal numbers of each subtype.

### 5.10 Immunohistochemistry of Axon Initial Segment

Following completion of patching on a retinal flatmount, the tissue was removed from the perfusion chamber and immediately fixed in paraformaldehyde 4% in 1× phosphate buffered saline (PBS) at 4°C for 30 min. The tissue was then washed 4–5 times in 0.1 M phosphate buffer (PB) and stored at 4°C in 0.1 M PB. Retinal flatmounts were incubated for 3 h at room temperature in a blocking solution of 10% normal goat serum (NGS) (Sigma‐Aldrich, USA) and 2% Triton X‐100 (Sigma‐Aldrich, Saint Louis, MO, USA). Retinas were then incubated in mouse monoclonal AnkyrinG antibody (1:500, Neuromab, USA) or rabbit polyclonal Ankyrin G antibody (1:250, Santa Cruz Biotechnology, USA) and chicken βIV‐spectrin antibody (1:100, Gift from M. Rasband, Baylor College of Medicine, TX, USA) (Susuki et al., 2012) for 3 nights at 4°C in PB containing 0.5% Triton X‐100, 3% NGS and 0.05% ProClin 300 (Sigma‐Aldrich, USA). Retinas were washed in PB, before being further incubated with mouse βIII‐tubulin antibody (1:500, Covance, USA). Retinas were then washed in PB and incubated in PB containing 0.5% Triton X‐100 for 4 h at 4°C with corresponding secondary antibodies including goat anti‐chicken Alexa 488, goat anti‐rabbit Alexa 594, goat anti‐chicken Alexa 594 and goat anti‐mouse Alexa 647 (all 1:300, Thermofisher Scientific, VIC, Australia). Retinas were then washed, labeled with DAPI (4′,6‐diamidino‐2‐phenylindole) and mounted ganglion cell layer up onto glass slides in Prolong Gold Antifade Mountant (Thermofisher Scientific, VIC, Australia).

#### 5.10.1 Imaging and Analysis

Three‐dimensional tiled image stacks of the ganglion cell and RNFL in the mid‐peripheral retina were created on a Zeiss LSM‐780 using a Plan‐Apochromat 63X/1.4 NA Oil DIC objective (Zeiss, Germany) 1.5× magnification and frame size 1024 × 1024 at resolution satisfying Nyquist sampling (XY resolution 0.088 nm, Z‐step interval 0.400 nm). Image stacks were deconvolved using Huygens Professional software (Scientific Volume Imaging B.V, Hilversum, The Netherlands) and Neurolucida software was used to measure AIS length and location. An AIS was defined as a region of contiguous staining labeled with βIV‐spectrin or AnkyrinG. AIS length was measured between proximal and distal endpoints through the 3D space, with the endpoints defined as the disruption to contiguous staining by > 5 pixels or > 1 z slice. Location was defined as the distance along the axon from the edge of soma to the start of the AIS (Harty et al. 2013). This was measured where an AIS was clearly associated with an RGC axon labeled by βIII‐tubulin, and the axon unambiguously originated from an identifiable RGC soma. AISs were only included for analysis if a clearly defined start and endpoint was within the stack; the AIS was within the lower boundary of the RGC layer as defined by DAPI or βIII‐tubulin; and the AIS was unambiguously distinct from other AISs. Tracing was conducted manually by a single investigator masked to group allocation. These tracings were exported and analyzed using Neuroexplorer software (11.06.2, MBF Bioscience, VT, USA).

### 5.11 Statistical Analysis

Normality of data was assessed with the D'Agostino‐Pearson test. Datasets that did not meet the criteria for normal distribution were analyzed using the non‐parametric Kruskal‐Wallis test with Dunn's multiple comparisons post hoc test. Parametric data were analyzed by either (i) a one‐way ANOVA with a Dunnett's post hoc test for comparisons to a control group or a Tukey–Kramer post hoc test for multiple comparisons or (ii) a two‐way ANOVA with Tukey's HSD post hoc test.

## Author Contributions

J.G.C. and S.P. conceived the project and secured funding. V.C., S.E., L.E.F., R.J.H., and E.T.F. planned and performed animal experiments and analyzed data. J.G.C., S.P., Pv.W., I.A.T., K.C.B., and V.C. assisted with experimental design, technical advice and guidance on data analysis. V.C. drafted the manuscript. All authors read and reviewed the manuscript.

## Conflicts of Interest

The authors declare no conflicts of interest.

## Data Availability

The data that support the findings of this study are available on request from the corresponding author. The data are not publicly available due to privacy or ethical restrictions.
